# Optimal use of anti-EGFR monoclonal antibodies for patients with advanced colorectal cancer: a meta-analysis

**DOI:** 10.1007/s10555-017-9668-y

**Published:** 2017-07-10

**Authors:** E. J. van Helden, C. W. Menke-van der Houven van Oordt, M. W. Heymans, J. C. F. Ket, R. van den Oord, H. M. W. Verheul

**Affiliations:** 1 Department of Medical Oncology, Cancer Center Amsterdam, VU University Medical Center, Amsterdam, The Netherlands; 2 Department of Epidemiology and Biostatistics, VU University Medical Center, Amsterdam, The Netherlands; 3 VU University, Medical Library, De Boelelaan 1117 (ZH3A-46), Postbus 7057, Amsterdam, 1081 HV The Netherlands

**Keywords:** Colorectal cancer, Anti-EGFR monoclonal antibodies, Meta-analysis, Treatment response, Progression-free survival, Overall survival

## Abstract

This meta-analysis was performed to determine the optimal use of anti-EGFR mAb in the treatment of metastasized colorectal cancer (mCRC). Seventeen randomized clinical trials were included, all evaluating the added value of anti-EGFR mAb to standard treatment line in patients with KRAS wild-type mCRC. Hazard and odds ratios were pooled using a random effect model, weighted according to cohort size. Pooled data of six first- and two second-line studies demonstrated a significantly improved ORR (OR 1.62, CI 1.27–2.04; OR 4.78, CI 3.39–6.75, respectively) and PFS (HR 0.79, CI 0.67–0.94; HR 0.80, CI 0.71–0.91, respectively) with the addition of anti-EGFR mAb to chemotherapy, while OS remained similar. Two third-line anti-EGFR mAb monotherapy studies revealed an improved PFS and OS (HR 0.44, CI 0.35–0.52; HR 0.55, CI 0.41–0.74). Addition of anti-EGFR *versus* anti-VEGF mAb to first-line chemotherapy was evaluated in three studies; ORR and PFS were comparable, while OS was improved (HR 0.8, CI 0.65–0.97). The influence of the chemotherapy backbone on anti-EGFR mAb efficacy, evaluated with meta-regression, indicated a higher ORR with irinotecan-based *versus* oxaliplatin-based regimens, but comparable PFS and OS. Reported toxicity (≥3 grade) increased ~20% in all treatment lines with the addition of anti-EGFR mAb. Anti-EGFR treatment significantly improves response and survival outcome of patients with (K)RAS wild-type mCRC, regardless of treatment line or chemotherapeutic backbone. Saving anti-EGFR mAb as third-line monotherapy is a valid and effective option to prevent high treatment burden caused by combination therapy. Combination treatment with anti-EGFR mAb to achieve radical resection of metastases needs further investigation.

**Electronic supplementary material:**

The online version of this article (doi:10.1007/s10555-017-9668-y) contains supplementary material, which is available to authorized users.

## Introduction

Worldwide colorectal cancer is the second most common cancer in women and the third in men [1]. Irresectable, non-curable colorectal cancer can be treated with palliative chemotherapy to reduce cancer symptoms, improve quality of life, and overall survival. In 1963, Heidelberger et al. discovered 5-fluorouracil (5-FU) as the first systemic chemotherapy for colorectal cancer. To date, this is the most effective and widely used systemic treatment for colorectal adenocarcinoma. In the course of time, combinations of fluoropyrimidines, with oxaliplatin and irinotecan, as well as the use of anti-EGFR and anti-VEGF targeted agents have improved survival of patients with metastasized colorectal cancer (mCRC) to about 2.5 years [2].

Cetuximab, a chimeric human and mouse monoclonal antibody (mAb), and panitumumab, a fully human mAb, both bind the epidermal growth factor receptor (EGFR). This prevents activation of the intracellular EGFR tyrosine kinase, resulting in an inhibition of the associated downstream signaling pathways, such as the RAS-RAF-MAPK and the PI3K-PTEN-AKT axis [3]. Additionally, antibody-dependent cell-mediated cytotoxicity (ADCC) may play a role in the efficacy of anti-EGFR mAb.

In 2008, a retrospective analysis revealed that the presence of mutations in Kirsten rat sarcoma viral oncogene homolog (KRAS) exon 2 has a negative predictive value for benefit from anti-EGFR therapy [4, 5]. Recently, the same was demonstrated for mutations in KRAS exons 3 and 4, and for the rare mutations in neuroblastoma rat sarcoma (NRAS) viral oncogene homolog exon 2–4 [6]. Despite patient selection based on wild-type (WT) RAS status, approximately 30% will not have clinical benefit from anti-EGFR mAb treatment [7]. Therefore, additional predictive biomarkers are needed.

In multiple clinical trials, the efficacy of anti-EGFR mAb has been evaluated as monotherapy or combined with different types of chemotherapy in patients with mCRC. Yet, the optimal sequence and combination for the use of anti-EGFR therapy remains unclear. With this meta-analysis, we aim to get more insight in the optimal clinical strategy for the use of anti-EGFR therapy. All included randomized controlled clinical trials in a KRAS WT mCRC population compared the additional benefit of anti-EGFR mAb therapy to first- or second-line chemotherapy treatment or to best supportive care in third-line treatment. We pooled efficacy data to objectify and compare overall response rate (ORR), progression-free survival (PFS), and overall survival (OS) for each treatment line. With meta-regression, the influence of the chemotherapeutic backbone and type of anti-EGFR mAb were analyzed. Furthermore, we evaluated whether the addition of anti-EGFR mAb is superior to anti-VEGF mAb in first-line treatment.

## Methods

### Search

A review protocol was developed based on the Preferred Reporting Items for Systematic Reviews and Meta-Analysis (PRISMA) statement (www.prisma-statement.org). PubMed, Embase.com, and Wiley/Cochrane Library were searched from inception (by EvH and JCFK) up to 17 February 2016. Hereafter, the search was repeated weekly to evaluate new potential records. The following terms were used (including synonyms and closely related words) as index terms or free-text words: “colorectal neoplasms” and “cetuximab” and “RCT” and “survival.” Studies were selected using predefined inclusion criteria: randomized controlled trial, evaluation of efficacy (OS, PFS, and ORR) of anti-EGFR monoclonal antibodies, and KRAS WT (at least exon 2) population. Studies were screened and selected by two independent reviewers (EvH and RvO) using Reference Manager (version 12.0.3 Thomson Reuters). Risk for potential bias was assessed using the Cochrane collaboration’s tool (EvH and RvO). The full search strategies for all the databases and all used inclusion and exclusion criteria to screen for relevant articles can be found in the Supplementary Information 1. All languages were accepted. Duplicate articles were excluded.

### Statistics

Hazard ratios (HRs) with standard errors (SEs) or confidence intervals (CIs) for PFS and OS were extracted from included studies. For ORR, odds ratios (ORs) with SE and CI were extracted. If the ORs were not stated in the publication, it was calculated from the percentage ORR and sample size if possible. SPSS (version 22, IBM Corp., Armonk, NY) was used for data entry; statistical analysis of the data was done in STATA version 12. A meta-analysis with a random effect model was used to generate a pooled summary effect size. All studies were weighted according to the number of included patients.

Heterogeneity between studies was visually evaluated using forest plots (non-overlapping confidence intervals indicate potential heterogeneity). To clarify potential heterogeneity between studies, meta-regression was used to test for different variables, such as chemotherapeutic backbone (5-FU, capecitabine, oxaliplatin, or irinotecan), type of anti-EGFR mAb (cetuximab or panitumumab), and summed points of the Cochrane collaboration’s tool. Differences with a *p* value <0.05 were considered relevant.

## Results

**Fig. 1:**
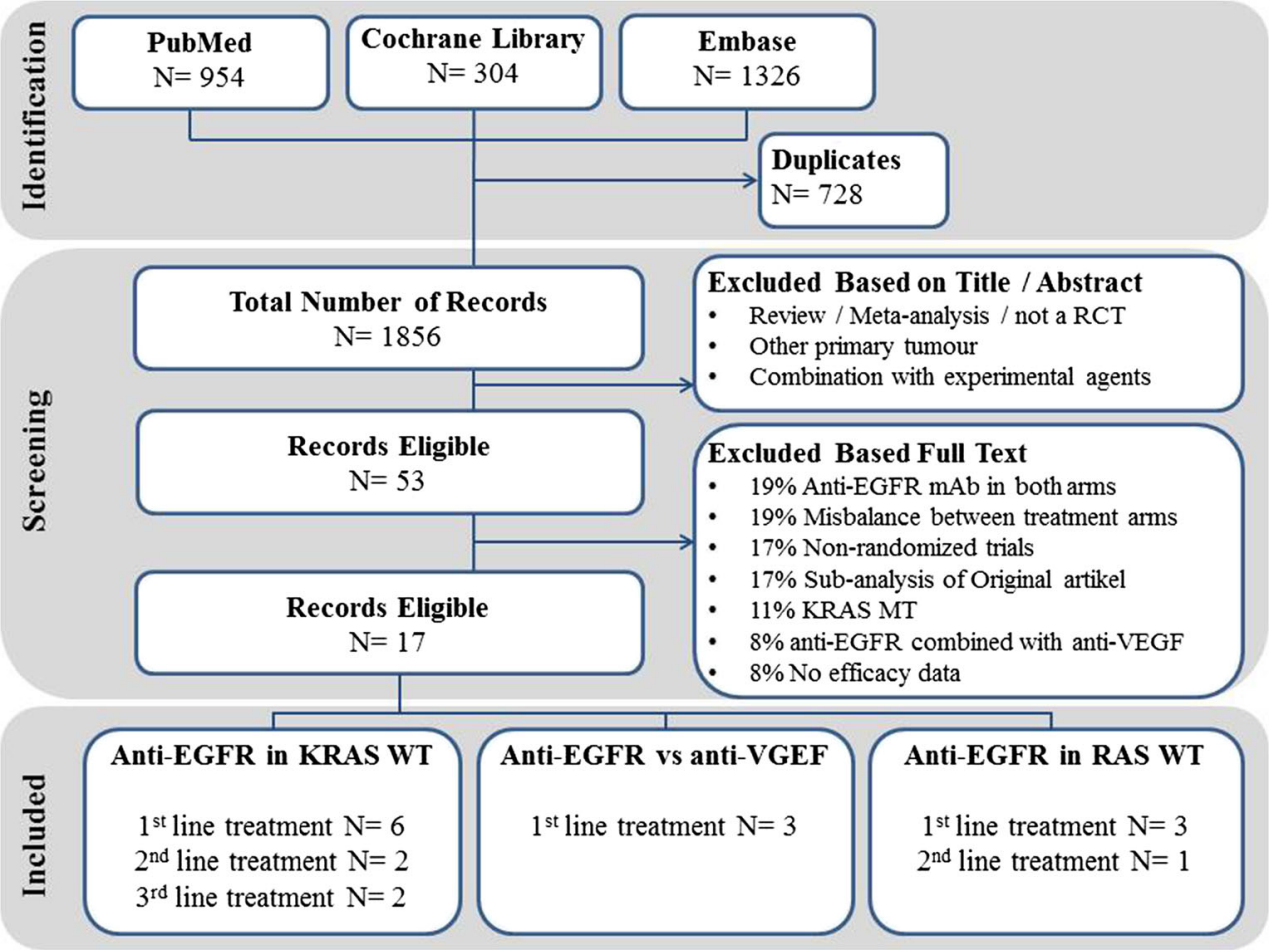
ᅟIdentification, screening, and included records

With our literature search, 1856 records were obtained; 1803 records did not meet the inclusion criteria based on title and abstract (Fig. 1). Of the remaining 53 records, 37 records were excluded based on full text review. Reasons for exclusion were anti-EGFR mAb in both arms (19%), a misbalance between treatment arms (19%), non-randomized trials (17%), sub-analysis of an original article (17%), KRAS-mutated population (11%), the combination of anti-EGFR mAb with anti-VEGF mAb (8%), and no reported efficacy data (8%) (Fig. 1). The 17 included publications are summarized in Tables 1 and 2. Pooled analyses were done for six first-line studies (*n* = 2580 patients), two second-line studies (*n* = 1057), and two third-line studies (*n* = 444). Of these trials, four studies published RAS WT data (*n* = 1464 patients), which were pooled. Additionally, three first-line studies that compared anti-EGFR mAb with anti-VEGF mAb were pooled (*n* = 2014 patients). Risk of bias was assessed using the Cochrane collaboration’s for randomized trials; all studies had a fairly low risk for bias (Supplementary Fig. 1).

**Table 1:** ᅟSummary of included publications

A. The addition of an anti-EGFR mAb to the first-line treatment of mCRC					
Study (author)	Combination (no. pt)	Control (no. pt)	Response rate % (OR, CI, *p*)	Median PFS (months) (HR, CI, *p*)	Median OS (months) (HR, CI, *p*)
OPUS (Bokemeyer)	Cetux + FOLFOX4 (82)	FOLFOX4 (97)	57 *versus* 34 (2.6, 1.4–4.7, 0.003)	8.3 *versus* 7.2 (0.57, 0.4–0.9, 0.006)	22.8 *versus* 18.5 (0.894, 0.6–1.2, 0.56)
CRYSTAL (v Cutsem)	Cetux + FOLFIRI (316)	FOLFIRI (350)	57 *versus* 40 (2.1, 1.5–2.8, <0.001)	9.9 *versus* 8.4 (0.7, 0.6–0.9, 0.001)	23.5 *versus* 20.0 (0.8, 0. 7–1.0, 0.009)
NORDIC-VII (Tveit)	Cetux + Nordic FLOX (97)	Nordic FLOX (97)	–	7.9 *versus* 8.7 (1.07, 0.8–1.5, 0.66)	20.1 *versus* 22.0 (1.14, 0.8–1.61, 0.48)
– (Ye)	Cetux + FOLFIRI or mFOLFOX6 (70)	FOLFIRI or mFOLFOX6 (86)	57 *versus* 29 (2.1, 1.1–4.1, 0.02)	10.2 *versus* 5.8 (0.6, 0.4–0.9, 0.004)	30.9 *versus* 21.0 (0.54, 0.3–0.9, 0.013)
MRC COIN (Maughan)	Cetux + FOLFOX/CAPOX (362)	FOLFOX/CAPOX (367)	64 *versus* 57 (1.4, 1.0–1.8, 0.049)	8.6 *versus* 8.6 (0.96, 0.8–1.1, 0.6)	17.0 *versus* 17.9 (0.96, 0.8–1.2, 0.67)
PRIME (Douillard)	Pani + FOLFOX4 (325)	FOLFOX4 (331)	57 *versus* 48 (1.5, 1.1–2.0, 0.02)	10.0 *versus* 8.6 (0.8, 0.6–1.0, 0.01)	23.9 *versus* 19.7 (0.88, 0.7–1.1, 0.17)
B. The addition of an anti-EGFR mAb *versus* an anti-VEGF mAb to the first-line treatment of mCRC					
CALGB/SWOG 80405 (Vernook)	Cetux + FOLFOX or FOLFIRI (578)	Beva + (FOLFOX or FOLFIRI) (559)	–	10.4 *versus* 10.8 (1.04, 0.9–1.2, 0.55)	29.9 *versus* 29.0 (0.92, 0.78–1.09, 0.34)
FIRE-3 (Heinemann)	Cetux + FOLFIRI (297)	Beva + FOLFIRI (295)	62 *versus* 58 (1.2, 0.9–1.6, 0.18)	10.0 *versus* 10.3 (1.06, 0.9–1.3, 0.55)	28.7 *versus* 25.0 (0.77, 0.62–0.96, 0.017)
PEAK (Schwartsberg)	Pani + mFOLFOX6 (142)	Beva + mFOLFOX6 (143)	58 *versus* 54 (1.1, 0.7–1.8, 0.59)	10.9 *versus* 10.1 (0.87, 0.7–1.2, 0.35)	34.2 *versus* 24.3 (0.62, 0.44–0.89, 0.009)
C. The addition of an anti-EGFR mAb to the second-line treatment of mCRC					
20,050,181 (Peeters)	Pani + FOLFIRI (303)	FOLFIRI (294)	36 *versus* 10 (5.5, 3.3–8.9, <0.001)	6.7 *versus* 4.9 (0.82, 0.7–1.0, 0.02)	14.5 *versus* 12.5 (0.92, 0.8–1.1, 0.37)
PICCOLO (Seymour)	Pani + irinotecan (230)	Irinotecan (230)	34 *versus* 12 (4.1, 2.5–6.8, <0.001)	5.5 *versus* 4.7 (0.78, 0.6–1.0, 0.02)	10.4 *versus* 10.9 (1.01, 0.83–1.23, 0.91)
D. The addition of an anti-EGFR mAb to the third-line treatment of mCRC					
20,020,408 (Amado)	Pani + BSC (115)	BSC (114)	17 *versus* 0	3.1 *versus* 1.8 (0.45, 0.3–0.6, <0.001)	8.1 *versus* 7.6 (0.99, 0.8–1.3)^a^
CO.17 (Karapetis)	Cetux +BSC (110)	BSC (105)	13 *versus* 0	3.7 *versus* 1.9 (0.4, 0.3–0.5, <0.001)	9.5 *versus* 4.8 (0.55, 0.4–0.7, <0.001)

*mAb* monoclonal antibodies, *mCRC* metastatic colorectal cancer, *Cetux* cetuximab, *Pani* panitumumab, *Beva* bevacizumab, *BSC* best supportive care, *OR* odds ratio, *CI* confidence interval, *HR* hazard ratio, *OS* overall survival, *PFS* progression-free survival

^a^Crossover design

**Table 2:** Summery of included RAS WT publications

Study (author, date)	Treatment line	Combination (no. pt)	Control (no. pt)	Response rate % (OR, CI, *p*)	Median PFS (months) (HR, CI, *p*)	Median OS (months) (HR, CI, *p*)
OPUS (Bokemeyer, May 2014)	First	Cetux + FOLFOX4 [8]	FOLFOX4 (49)	58 *versus* 29 (3.3, 1.4–8.2, 0.008)	12.0 *versus* 5.8 (0.53, 0.3–1.0, 0.06)	19.8 *versus* 17.8 (0.94, 0.6–1.6, 0.8)
PRIME (Douillard, September 2013)	First	Pani + FOLFOX4 (259)	FOLFOX4 (253)	–	10.1 *versus* 7.9 (0.72, 0.58–0.90, 0.004)	25.8 *versus* 20.2 (0.77, 0.64–0.94, 0.009)
CRYSTAL (v Cutsem, January 2015)	First	Cetux + FOLFIRI (178)	FOLFIRI (189)	61 *versus* 38 (2.64, 1.78–3.92, 0.001)	11.3 *versus* 7.1 (0.58, 0.44–0.77, 0.001)	26.1 *versus* 20.2 (0.75, 0.60–0.93, 0.008)
20,050,181 (Peeters, December 2015)	Second	Pani + FOLFIRI (204)	FOLFIRI (294)	–	6.4 *versus* 4.4 (0.70, 0.54–0.90, 0.006)	16.2 *versus* 13.9 (0.80, 0.63–1.0, 0.077)

*mCRC* metastatic colorectal cancer, *Cetux* cetuximab, *Pani* panitumumab, *BSC* best supportive care, *OR* odds ratio, *CI* confidence interval, *HR* hazard ratio, *OS* overall survival, *PFS* progression-free survival

### First-line treatment

**Fig. 2:**
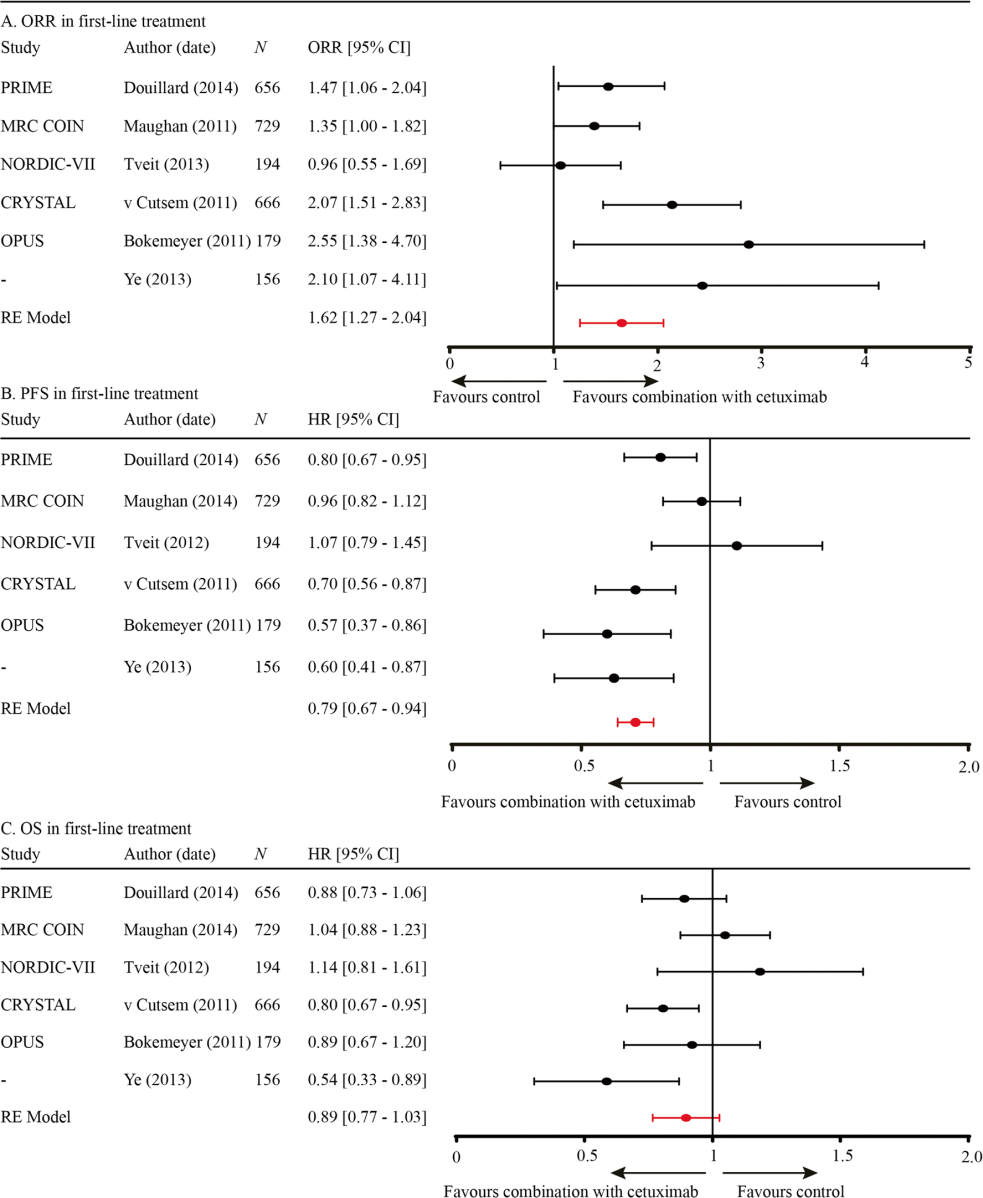
**a** ORR in first-line treatment. **b** PFS in first-line treatment. **c** OS in first-line treatment

Of all publications included in this meta-analysis, six evaluated chemotherapy with and without anti-EGFR mAb in the first line [9–14]. Pooled data revealed that ORR and PFS significantly improved by the addition of anti-EGFR mAb treatment (OR 1.62, CI 1.27–2.07; HR PFS 0.79, CI 0.67–0.94; Fig. 2a, b, respectively), while OS did not improve (HR OS 0.89, CI 0.77–1.03; Fig. 2c).

Using meta-regression, the effect of the chemotherapeutic backbone on efficacy data was evaluated in all six first-line studies. In two out of six studies, the chemotherapeutic backbone was irinotecan-based; in the remaining four studies, it was oxaliplatin-based. Although there was a beneficiary trend for the irinotecan-based combination, PFS and OS were not significantly different between these groups (*p* = 0.09 and *p* = 0.06, respectively). The ORR was significantly higher for the studies combining anti-EGFR mAb with irinotecan *versus* oxaliplatin (OR 2.07 *versus* 1.42; *p* = 0.04). Besides a subgroup in the MRC COIN study, none of the included studies used capecitabine as fluoropyrimidine backbone.

From three of the six first-line studies, retrospective analyses of RAS WT data were published [15–17]. Pooled analyses indicated that ORR, PFS, and OS were significantly improved with the addition of anti-EGFR mAb (OR 2.74, CI 1.91–3.94; HR 0.65, CI 0.55–0.77; HR 0.77, CI 0.67–0.89, respectively).

### Second-line treatment

In two studies, second-line chemotherapy with or without anti-EGFR mAb was compared [18, 19]. Comparable to first-line studies, ORR and PFS were significantly improved in the arms that included anti-EGFR mAb (OR 4.78, CI 3.39–6.75; HR 0.80, CI 0.71–0.91). OS remained unaffected (HR 0.96, CI 0.84–1.10). In the 20,050,181 study, 45.5% of the patients in the FOLFIRI alone arm received anti-EGFR mAb therapy after progression; this could reduce the observed benefit in OS in the combination arm [19]. In the PICCOLO study, only 6% of the control group received subsequent anti-EGFR mAb therapy and data concerning other subsequent therapies were not collected [18].

### Anti-EGFR mAb monotherapy

Two monotherapy studies compared anti-EGFR mAb monotherapy *versus* best supportive care in chemotherapy-refractory patients with mCRC. ORR was not evaluable using ORs, as none of the patients in the best supportive care (BSC) arm had a response. The 20,020,408 [4] and the CO.17 study [5] reported an ORR of 17 and 13%, respectively, in patients treated with anti-EGFR monotherapy. Pooled data demonstrated a significantly longer PFS in the arm with anti-EGFR therapy (HR 0.44, CI 0.35–0.52). The 20,020,408 study had a crossover design; therefore, OS is not comparable. Karapetis et al. reported that overall survival doubled (9.5 *versus* 4.8 months) with the addition of anti-EGFR mAb to best supportive care (HR 0.55, CI 0.41–0.74) [5].

### Wild-type RAS

Of all included studies in which the added benefit of anti-EGFR to chemotherapy was evaluated, one second-line and three first-line studies retrospectively assessed the effect in a RAS WT group (*n* = 1464 patients), excluding patients whose tumor harbored additional mutations in KRAS exon 3–4 and NRAS exon 2–4 [15–17, 20]. All efficacy data of the combination arm improved compared to the KRAS exon 2 WT group (ORR OR 2.74 and PFS HR 0.67). In the RAS WT group, overall survival was significantly improved with the addition of an anti-EGFR mAb, with a HR of 0.78 (CI 0.69–0.88) (Table 2).

### Chemotherapeutic backbone

In six first-line and two second-line studies, chemotherapy with or without anti-EGFR mAb in patients with KRAS WT mCRC were evaluated. Pooled efficacy data of these eight studies demonstrated an improved ORR (OR 2.14, CI 1.47–3.12) and PFS (HR 0.8, CI 0.71–0.9). There was no benefit observed for OS (HR 0.92, CI 0.83–1.03).

Meta-regression of first- and second-line studies demonstrated that for the irinotecan-based group, the addition of anti-EGFR mAb rendered a significantly higher ORR with an OR of 3.41 *versus* 1.45 in the oxaliplatin-based group (*p* = 0.002). PFS and OS did not significantly differ between the irinotecan- or oxaliplatin-based groups with the addition of anti-EGFR mAb (*p* = 0.10 and *p* = 0.51, respectively).

### Anti-EGFR *versus* anti-VEGF mAb in first-line treatment

**Fig. 3:**
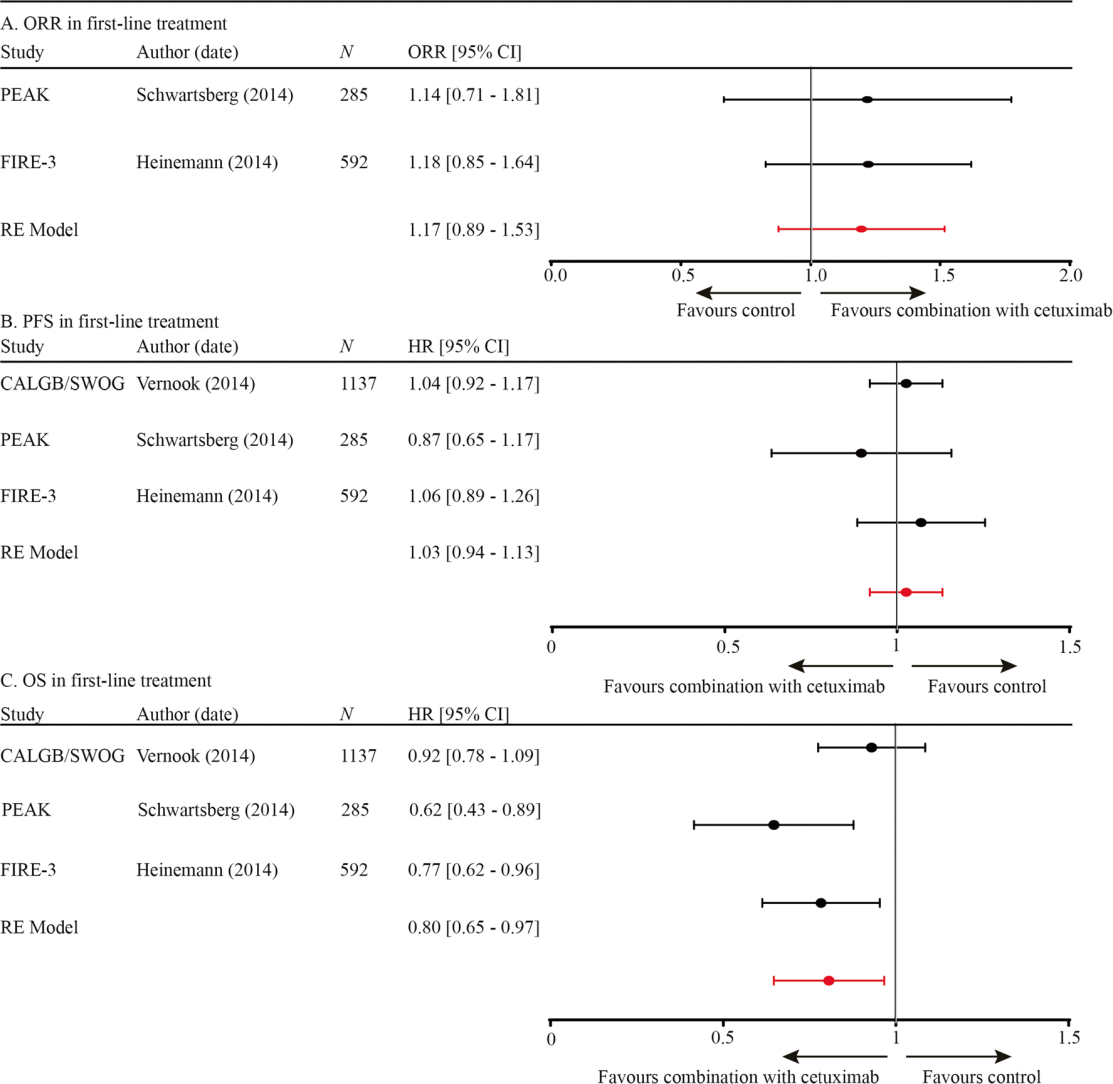
**a** ORR in first-line treatment. **b** PFS in first-line treatment. **c** OS in first-line treatment

Three randomized controlled trials evaluated the addition of anti-EGFR mAb or anti-VEGF mAb to first-line palliative chemotherapy [2, 21, 22]. The PEAK and the FIRE-3 studies revealed similar overall response rates of about 50–60% in both arms. Pooled ORR data were also equal between the two arms (OR 1.17, CI 0.89–1.53). Furthermore, PFS was similar for both arms in all three studies; pooled data demonstrated the same results (HR 1.03, CI 0.94–1.13; Fig. 3). OS was significantly improved for the anti-EGFR mAb arm in the PEAK and FIRE-3 studies with a HR of 0.62 and 0.77, respectively. In the large CALGB/SWOG 80405 study, there was a beneficiary trend towards the anti-EGFR mAb arm, but the difference in OS was not significant (HR 0.92, CI 0.78–1.09). Pooled data revealed an overall survival benefit with a HR of 0.80 (CI 0.65–0.97; Fig. 3). Based on the forest plots, no obvious heterogeneity was observed between the three studies; therefore, meta-regression was not done.

### Toxicity

Another consideration for the addition of an anti-EGFR mAb to chemotherapy is its potential additive toxicity. In Table 3, the percentage of grade ≥3 adverse events are listed for all included studies. As expected, anti-EGFR mAb-specific adverse events, such as (acneiform) rash, diarrhea, hypomagnesaemia, and infusion-related reaction, occurred more often in the combination groups. Adverse events such as anemia, thrombocytopenia, leucopoenia, neutropenia, fatigue, and palmar-plantar erythrodysesthesia were comparable between the two arms, probably since these adverse events were more likely to be caused by the chemotherapeutic backbone. In first-line treatment, any reported grade ≥3 adverse events occurred in 82% of all included patients in the combination arm *versus* 62% in the control arm. For the second line, these percentages were 67 *versus* 46%, and in third line, it was 58 *versus* 40% for best supportive care. Thus, the addition of anti-EGFR mAb in all treatment lines resulted in an absolute increase of grade ≥3 adverse events of approximately 20%.

**Table 3:** Percentage of grade ≥3 adverse events listed for all included studies

			(Acneiform) rash	Diarrhea	Anemia	Thrombocytopenia	Leucopoenia	Neutropenia	Hypomagnesaemia	Fatigue (lethargy)	(Peripheral) neuropathy	Palmar-plantar erythrodysesthesia	Infusion-related reactions	Any
First-line treatment	OPUS	Combination	18	9	4	3	7	35	–	1	14	4	2	82
		Control	0	5	2	0	5	32	–	3	7	1	1	64
	CRYSTAL	Combination	21	16	–	–	25	31	–	14	–	–	5	81
		Control	<1	10	–	–	17	24	–	20	–	–	0	60
	NORDIC-VII	Combination	22	17	1	4	14	46	–	16	23	–	7	–
		Control	1	10	1	3	21	47	–	10	32	–	3	–
	Ye et al.	Combination	13	6	–	–	11		–	–	4	–	3	–
		Control	3	4	–	–	9		–	–	6	–	2	–
	MRC COIN	Combination	20	24	5	3	4	13	4	26	14	10	–	–
		Control	1	14	2	3	4	12	0	18	18	4	–	–
	PRIME	Combination	37	18	–	–	–	43	7	10	–	–	<1	–
		Control	2	9	–	–	–	42	<1	3	–	–	0	–
Mean toxicity first line		Combination	22	15	3	3	12	34	6	13	14	7	4	82
		Control	1	9	2	2	11	31	0	11	16	3	1	62
Second line	20,050,181	Combination	37	14	–	–	–	20	3	–	–	–	<1	73
		Control	2	9	–	–	–	23	1	–	–	–	0	52
	PICCOLO	Combination	19	30	4	2	–	22	–	21	–	–	–	61
		Control	0	19	1	0	–	12	–	11	–	–	–	39
Mean toxicity second line		Combination	28	22	4	2	–	21	3	21	–	–	<1	67
		Control	1	14	1	0	–	18	1	11	–	–	0	46
Third line	20020408^a^	Combination	8	1	–	–	–	–	–	4	–	–	–	37
		Control	0	0	–	–	–	–	–	3	–	–	–	20
	CO.17^b^	Combination	34	–	–	–	–	–	15	33	–	–	13	79
		Control	1	–	–	–	–	–	0	26	–	–	0	59
Mean toxicity third line		Combination	21	1	–	–	–	–	15	19	–	–	13	58
		Control	1	0	–	–	–	–	0	15	–	–	0	40
Anti-VEGF *versus* anti-EGFR agent	C/S 80405	Anti-EGFR	–	–	–	–	–	–	–	–	–	–	–	–
		Anti-VEGF	–	–	–	–	–	–	–	–	–	–	–	–
	FIRE-3	Anti-EGFR	26	11	<1	–	<1	<1	4	<1	0	3	4	–
		Anti-VEGF	2	14	0	–	0	0	1	1	<1	<1	0	–
	PEAK	Anti-EGFR	32	–	–	1	–	–	7	11	–	–	–	63
		Anti-VEGF	1	–	–	0	–	–	0	9	–	–	–	56

^a^Based on original KRAS unselected group v Cutsem et al. JCO 2007

^b^Based on original KRAS unselected group Jonker et al. NEJM 15 2007

### Proposed criteria to evaluate optimal use of anti-EGFR mAb

#### Differences in (progression-free) survival between treatment lines

The addition of anti-EGFR mAb in first- or second-line treatment renders the same beneficiary effect (first-line HR 0.79 *versus* second-line HR 0.80). The HR of PFS in the third line is not comparable to first or second line as it is compared to BSC.

OS in first and second line for the KRAS WT population was similar between the combination arm *versus* the control arm. Yet, in the RAS WT group, a significant improvement was seen in first-line treatment (HR of 0.77, CI 0.67–0.89). Only one second-line study, 20,050,181, reported survival in RAS WT data, with a non-significantly different survival between the two arms (median OS combination 16.2 *versus* 13.9 months, HR of 0.80, *p* = 0.08) [20]. OS in the third line was only evaluable in the CO.17, which revealed an improved OS with a HR of 0.55 (*p* < 0.001).

#### Differences in efficacy data due to the chemotherapeutic backbones

Between the included first- and second-line studies, ORR, PFS, and OS for combinations with irinotecan *versus* oxaliplatin were compared using meta-regression. ORR was significantly different, with an OR of 3.41 in the irinotecan combinations *versus* an OR of 1.45 in the oxaliplatin combinations (*p* = 0.0016). However, this benefit for irinotecan combinations was not reflected by PFS and OS gain (*p* = 0.10 and *p* = 0.51, respectively).

#### Differences in toxicity between treatment lines

In all treatment lines, there was an added absolute incidence of grade ≥3 adverse events of approximately 20% with the addition of anti-EGFR mAb. The total incidence of any grade ≥3 adverse events was 82% in the first-line combination therapy group, while this was 58% in third-line setting.

## Discussion

The aim of this meta-analysis was to define the optimal use of anti-EGFR mAb in the non-curative treatment of mCRC. To determine its optimal use, we evaluated benefit (e.g., ORR, PFS, and OS) and toxicity that resulted from anti-EGFR mAb addition to standard chemotherapy or as monotherapy. Additionally, we assessed the influence of the treatment line as well as the chemotherapeutic backbone on efficacy of anti-EGFR mAb.

To determine optimal treatment for patients with non-curative mCRC, OS is the most important clinical outcome measure. However, benefit in OS is difficult to objectify due to the influence of subsequently applied systemic or, in some cases, local treatment strategies. Additionally, subsequent treatment data are often incompletely collected and reported. In the first- and second-line studies, there was no clear benefit in OS for the KRAS WT cohorts receiving anti-EGFR mAb therapy (HR 0.89, *p* = 0.13; HR 0.96, *p* = 0.54, respectively), but retrospective analyses in the RAS WT population indicated that the addition of anti-EGFR mAb to first-line chemotherapy significantly improved OS (HR 0.77, CI 0.67–0.89). In the third line, only the CO.17 trial provided a correct representation of benefit in OS with a HR of 0.55 (*p* < 0.001) compared to BSC. Benefit in PFS due to the addition of anti-EGFR mAb was comparable in first and second line (HR 0.79 and 0.80, respectively). In third line, the HR for PFS was 0.43. Yet, this greater effect of anti-EGFR mAb treatment is partly caused by the fact that it is compared to BSC, as the added median time to PFS with the addition of anti-EGFR mAb was comparable between all treatment lines. The last outcome measure, ORR, is often not paramount in a non-curative setting. An exception is the intent to convert unresectable to resectable disease, frequently a point of discussion for patients with unresectable liver-limited CRC metastases. In literature, the addition of anti-EGFR mAb to neo-adjuvant treatment has given contradictory results [23, 24]. Currently, a prospective multicenter RCT is including patients with liver-limited CRC metastases to further investigate the role of anti-EGFR mAb to convert irresectable to resectable disease [25]. In our study, pooled efficacy data revealed a significantly higher ORR in all treatment lines with the addition of an anti-EGFR mAb. However, based on the evaluated data, no obvious differences in added gain of OS, PFS, and ORR were demonstrated with the addition of anti-EGFR mAb in first-, second-, and third-line treatment to guide its optimal clinical use.

It has been suggested that the addition of an anti-EGFR mAb to irinotecan-based regimen has a synergetic effect, opposed to oxaliplatin-based regimens [26, 27]. With meta-regression, we have shown that anti-EGFR mAb rendered a significantly better ORR in the irinotecan-based regimen compared to the oxaliplatin-based regimen (*p* = 0.04). However, this superior effect was not confirmed for PFS and OS (*p* = 0.09 and *p* = 0.06, respectively). These results are in concordance with multiple randomized trials, which demonstrated no differences in treatment benefit for irinotecan and anti-EGFR mAb compared to oxaliplatin and anti-EGFR [8, 28–30].

The addition of either anti-EGFR mAb or anti-VEGF mAb to first-line non-curative chemotherapy is a much-debated current clinical issue. Pooled data of all available first-line studies, which compared the addition of both antibodies, demonstrated a comparable ORR and PFS, while OS was significantly longer in the anti-EGFR arm (HR of 0.8, CI 0.65–0.97). The benefit in OS was unexpected, as it is not in line with the other outcome measurements such as ORR and PFS. The significant benefit in OS was observed in two studies, the FIRE-3 [22] and the PEAK [21]. In the FIRE-3, ORR (the primary endpoint) as well as the PFS were similar in the cetuximab arm compared to the bevacizumab arm (62 *versus* 58% and 10.0 *versus* 10.3 months), while OS was 28.7 *versus* 25.0 months (*p* = 0.017, respectively) [22]. Although a recent post hoc analysis on centrally reviewed CT images reported a significant improvement with the addition of anti-EGFR mAb (72 *versus* 56%; *p* = 0.003), PFS remained similar between the anti-EGFR and the anti-VEGF mAb arms (8.4 *versus* 9.7 months; *p* = 0.53) [31]. The observed discrepancy between PFS and OS is most likely caused by small imbalances in subsequent treatments, such as more use of oxaliplatin and fluoropyrimidine in the anti-EGFR mAb arm [32]. Additionally, only 52% of the patients in the anti-VEGF mAb arm received anti-EGFR mAb treatment, compared to all patients in the anti-EGFR arm [32]. In fact, the PFS for the second-line therapy in the anti-EGFR mAb arm was significantly longer (HR 0.68, *p* < 0.001) [32], indicating that indeed the differences in OS were not caused by first-line treatment but by differences in later treatment. Perhaps, data of the patient distribution of left *versus* right sidedness in both arms might provide more insight in this discrepancy between ORR, PFS, and OS. For the smaller PEAK trial, no data concerning the subsequent treatment were published [21]. In the largest trial, which evaluated anti-EGFR mAb *versus* anti-VEGF mAb as addition to first-line treatment, the CALGB/SWOG 80405 trial, 1137 patients were treated with FOLFIRI and cetuximab or bevacizumab. No significant differences in PFS or OS were found between the two arms. Unfortunately, no full paper of this study has been published yet, precluding detailed analysis. In the second line, the SPIRITT trial is the only study that compared anti-EGFR with anti-VEGF mAb in addition to combination chemotherapy (FOLFIRI), after progression on first-line, oxaliplatin-based treatment with bevacizumab. As there are no other studies comparing these two agents in second line, we could not pool data. We did not pool this study with the three first-line studies, because of the difference in treatment setting. In the SPIRITT phase 2 trial, PFS and OS were similar for the two regimens [33]. It is remarkable that no difference was observed, because patients received bevacizumab in first line, probably not all patients were truly resistant to the first-line treatment regimen. Oxaliplatin toxicity may have influenced the switch to second-line treatment. Nevertheless, these data suggest that the addition of either mAb to this treatment setting improves clinical outcome.

It might be argued that first-line treatment is the most important treatment line, and responsible for the main gain in OS. This is the key rationale to add anti-EGFR mAb to first-line treatment. However, in the last decade, OS improved with 10 months, whereas PFS of the first-line treatment remained the same [34], indicating that the gain in OS most likely results from improved care for patients by using the total arsenal of available systemic treatments. In addition, multiple studies showed that survival benefit of combination therapy is comparable to sequential therapy, whereas combination therapy is significantly more toxic [35–40]. Indeed, in our meta-analysis, the occurrence of any ≥3 grade adverse events in first-line combination treatment was 82% compared to 64% with standard chemotherapy. Based on these data, using anti-EGFR mAb as third-line monotherapy is a sensible and tolerable treatment option after progression on standard (combination) chemotherapy.

In order to improve efficacy of anti-EGFR treatment, predictive biomarkers are urgently needed. An obvious biomarker, EGFR expression on tumor tissue, did not correlate with treatment benefit [8, 27–29]. A well-established biomarker that predicts primary resistance to anti-EGFR mAb are RAS mutations (KRAS and NRAS exon 2–4). This meta-analysis only included studies excluding patients with KRAS exon 2 mutated tumors, as these were the first and most common mutations known to induce resistance. Thus, our results are most likely an underestimation of the efficacy of anti-EGFR mAb treatment for the RAS wild-type cohort. Retrospective analyses of these additional RAS mutations were performed in four of the included studies [36]. Additional RAS mutations were confirmed in 14–26% of patients, resulting in improved efficacy data upon exclusion of these patients. A potential explanation for primary resistant patients with RAS wild-type mCRC is intralesional and interlesional heterogeneity in RAS mutation [41], making the RAS mutation determination on a single needle biopsy or old resection material prone for sampling errors. A promising novel approach is to evaluate these mutations in circulating cell-free DNA [42] or circulating tumor cells [43].

Recently, right-sided location of the primary tumor has been reported to negatively influence treatment benefit of anti-EGFR mAb [34, 44]. In the meta-analysis of Arnold et al., single patient data of six trials, which were also included in this meta-analysis, were pooled to evaluate the prognostic and predictive value of sidedness [45]. Indeed, they demonstrated that the addition of anti-EGFR mAb in patients with left-sided tumors significantly improved OS (HR = 0.75, CI 0.67–0.84) and PFS (HR = 0.78, CI 0.70–0.87), in contrast to patients with right-sided tumors (HR = 1.12, CI 0.87–1.45; HR = 1.12, CI 0.87–1.44, for OS and PFS, respectively). Further research is needed to evaluate the clinical utility of this biomarker and understand the underlying mechanisms of resistance.

## 4. Conclusion

Based on our meta-analysis, we conclude that the anti-EGFR treatment significantly improves response and survival outcome of patients with (K)RAS wild-type mCRC, regardless of treatment line or chemotherapeutic backbone. It is a sensible treatment strategy to save anti-EGFR mAb as third-line monotherapy for patients with mCRC in a true non-curative setting, as combination therapy is more toxic and has no clinically significant benefit compared to sequential therapy. For patients with limited disease, first-line combination therapy with anti-EGFR mAb can be considered, if local radical treatment may still be an option upon downstaging. As sound data to support this last consideration are lacking, further research is necessary.

## Electronic supplementary material

Supplementary Figure 1 (DOCX 14 kb)

ESM 1 (DOCX 18 kb)
