# Supplementary material for: Optimal use of anti-EGFR monoclonal antibodies for patients with advanced colorectal cancer: a meta-analysis
Source: Cancer Metastasis Rev. 2017 Jul 10;36(2):395–406. doi: 10.1007/s10555-017-9668-y (PMC5557867; doi:10.1007/s10555-017-9668-y)
Supplement: Supplementary file 2 — (DOCX 18 kb) [file 10555_2017_9668_MOESM2_ESM.docx]

# Supplemental Information 1. Search Protocol

**Search strategy for PubMed (17 February 2016)**

[Mesh] = Medical subject headings (MeSH)

[Supplementary Concept] = substance name from MeSH Database

[tiab] = words in title or abstract

[tw] = words in title, abstract and other fields, like MeSH

| **Search** | **Query** | **Items found** |
| --- | --- | --- |
| **#5** | #1 AND #2 AND #3 AND #4 | **954** |
| **#4** | "Survival Rate"[Mesh] OR survival*[tiab] OR os[tiab] OR pfs[tiab] OR disease control rate*[tiab] | **755,590** |
| **#3** | randomized controlled trial[pt] OR controlled clinical trial[pt] OR random*[tiab] OR placebo[tiab] OR drug therapy[sh] OR randomly[tiab] OR trial[tiab] OR groups[tiab] | **3,900,730** |
| **#2** | "cetuximab" [Supplementary Concept] OR "panitumumab" [Supplementary Concept] OR cetuximab[tiab] OR c225[tiab] OR erbitux[tiab] OR panitumumab[tiab] OR vectibix[tiab] OR abx erf*[tiab] | **5,470** |
| **#1** | "Colorectal Neoplasms"[Mesh] OR (("Neoplasms"[Mesh] OR carcinoma*[tw] OR neoplas*[tw] OR tumour*[tw] OR adenoma*[tw] OR tumor*[tw] OR cancer*[tw] OR cancer[sb] OR oncolog*[tw] OR malignan*[tw] OR metasta*[tw] OR carcinogen*[tw] OR oncogen*[tw] OR adenocarcinoma*[tw]) AND (colorectal*[tw] OR colon[tw] OR rectal*[tw] OR sigmoid*[tw] OR cecum*[tw] OR cecal*[tw] OR coecum*[tw] OR coecal*[tw])) | **261,547** |

**Search strategy for Embase.com (17 February 2016)**

/exp = EMtree keyword with explosion

:ab,ti = words in title or abstract

NEAR/x = words near to each other, x places apart

NEXT/1 = words next to each other, x places apart

| **Search** | **Query** | **Items found** |
| --- | --- | --- |
| **#5** | #1 AND #2 AND #3 AND #4 | **1,326** |
| **#4** | 'survival'/exp OR survival*:ab,ti OR os:ab,ti OR pfs:ab,ti OR ('disease control' NEAR/3 rate*):ab,ti | **1,155,214** |
| **#3** | random* OR factorial* OR crossover* OR cross NEXT/1 over* OR placebo* OR (doubl* AND blind*) OR (singl* AND blind*) OR assign* OR allocat* OR volunteer* OR 'crossover procedure'/exp OR 'double blind procedure'/exp OR 'randomized controlled trial'/exp OR 'single blind procedure'/exp | **1,882,940** |
| **#2** | 'cetuximab'/exp OR 'panitumumab'/exp OR cetuximab:ab,ti OR c225:ab,ti OR erbitux:ab,ti OR panitumumab:ab,ti OR vectibix:ab,ti OR (abx NEXT/1 erf*):ab,ti | **21,396** |
| **#1** | 'colorectal adenoma'/exp OR ('neoplasm'/exp OR carcinoma*:ab,ti OR neoplas*:ab,ti OR tumour*:ab,ti OR adenoma*:ab,ti OR tumor*:ab,ti OR cancer*:ab,ti OR oncolog*:ab,ti OR malignan*:ab,ti OR metasta*:ab,ti OR carcinogen*:ab,ti OR oncogen*:ab,ti OR adenocarcinoma*:ab,ti AND (colorectal*:ab,ti OR colon:ab,ti OR rectal*:ab,ti OR sigmoid*:ab,ti OR cecum*:ab,ti OR cecal*:ab,ti OR coecum*:ab,ti OR coecal*:ab,ti)) | **271,375** |

**Search strategy for Wiley/Cochrane Library (17 February 2016)**

ti,ab,kw = words in title, abstract or keyword

| **Search** | **Query** | **Items found** |
| --- | --- | --- |
| **#1** | ((carcinoma* or neoplas* or tumour* or adenoma* or tumor* or cancer* or oncolog* or malignan* or metasta* or carcinogen* or oncogen* or adenocarcinoma*) and (colorectal* or colon or rectal* or sigmoid* or cecum* or cecal* or coecum* or coecal*)):ti,ab,kw (Word variations have been searched) | **12,086** |
| **#2** | cetuximab or c225 or erbitux or panitumumab or vectibix or "abx erf" or "abx erfs":ti,ab,kw (Word variations have been searched) | **959** |
| **#3** | survival* or os or pfs or ("disease control" and rate*):ti,ab,kw (Word variations have been searched) | **52,575** |
| **#4** | #1 and #2 and #3 | **316** |

CENTRAL: 304

# In- and Exclusion Criteria

**Inclusion**

1. Studies concerning anti-EGFR monoclonal antibodies, which are clinically used as treatment for colorectal adenocarcinoma (e.g. Cetuximab or Panitumumab).
2. Main aim of the study is efficacy of treatment with anti-EGFR monoclonal antibodies in a palliative setting (first, second and third line treatment), with a primary outcome concerning ORR, OS or PFS.
3. Randomized controlled clinical trials (phase II or higher).
4. Included patients must be KRAS WT (at least exon 2), or the KRAS status was retrospectively determined and ORR, PFS and OS was specified for this selected subgroup.

**Exclusion**

1. Neoadjuvant or adjuvant setting.
2. Phase I articles or case reports or case series smaller than 10.
3. Clinical trials which include investigational (targeted) agents or procedures (such as radio-immuno therapy, TACE, brivanib or alaninate.
4. Trials with evaluate biomarkers / cost-effectiveness / management of adverse events of anti-EGFR MoAB as aim objective.
5. Articles that describe additional analyses of data of one cohort/study, most recent and complete publication will be selected for inclusion.
6. Other types of malignancies (also anal canal carcinoma).
7. Reviews or meta-analysis.
8. anti-EGFR MoAB in combination with bevacizumab, sorafenib or regorafenib.
